# Cep164, but not EB1, is critical for centriolar localization of TTBK2 and its function in ciliogenesis

**DOI:** 10.1186/2046-2530-4-S1-P66

**Published:** 2015-07-13

**Authors:** Toshiaki Oda, Shuhei Chiba, Tomoaki Nagai, Kensaku Mizuno

**Affiliations:** 1 Department of Biomolecular Sciences, Graduate School of Life Sciences, Tohoku University, Sendai, Miyagi 980-8578, Japan

## Objective

Primary cilia play crucial roles in sensing and transducing external signals. They extend the axoneme from the mother centriole-derived basal body. A centrosomal protein CP110 blocks axoneme extension and is removed from the mother centriole at the onset of ciliogenesis. TTBK2 is recruited to the mother centriole in response to serum starvation and is required for CP110 removal and ciliogenesis. To elucidate the mechanism of centriolar recruitment of TTBK2, we examined the roles of two TTBK2-binding proteins, EB1 and Cep164, in TTBK2 recruitment.

## Methods

The EB1- and Cep164-binding sites of TTBK2 were determined by co-precipitation assays, using their deletion and point mutants. The knockdown/rescue experiments of TTBK2 were performed using hTERT-RPE and IMCD3 cells.

## Results

TTBK2 interacts with EB1 via the SRIP and SKIP motifs in the C-terminal region of TTBK2 and the EBH domain of EB1. EB1 mediated TTBK2 localization at the plus-ends of microtubules but was not essential for its centriolar recruitment and ciliogenesis. TTBK2 interacts with Cep164 via the C-terminal proline-rich motif of TTBK2 and the WW domain of Cep164. The Cep164-non-binding mutant was not recruited to the centriole, and depletion of Cep164 suppressed centriolar recruitment of TTBK2. Thus, binding to Cep164 is required for centriolar recruitment of TTBK2. Knockdown/rescue experiments of TTBK2 revealed that wild-type TTBK2, but not Cep164-non-binding mutants, rescued CP110 removal and ciliogenesis in TTBK2-depleted cells. Thus, Cep164 binding of TTBK2 is critical for its function to promote CP110 removal and ciliogenesis. We also found that TTBK2 phosphorylates Cep164 and Cep97, and suppresses Cep164 binding to Dishevelled-3.

## Conclusion

Cep164, but not to EB1, is essential for centriolar localization of TTBK2 and its function in promoting CP110 removal and ciliogenesis. TTBK2 effectively phosphorylates Cep164 and Cep97.

